# Use of the A2DS2 scale to predict morbidity in stroke-associated pneumonia: a systematic review and meta-analysis

**DOI:** 10.1186/s12883-021-02060-8

**Published:** 2021-01-22

**Authors:** Jie Huang, Ming Liu, Weiliang He, Feifei Liu, Jinming Cheng, Hebo Wang

**Affiliations:** 1 North China University of Science and Technology, Tangshan, Hebei China; 2 Department of Neurology, Hebei General Hospital, 050000 Shijiazhuang, Hebei China; 3 Department of Global Health, School of Health Sciences, Wuhan University, Wuhan, Hubei China

**Keywords:** A2DS2 scale, Stroke‐associated pneumonia, Stroke, Meta‐analysis

## Abstract

**Background:**

This review aims to evaluate the performance and clinical applicability of the A2DS2 scale via systematic review and meta-analysis.

**Methods:**

The Medline, Embase, Cochrane Library, CBM, CNKI, and Wanfang databases were searched. The risk of bias was assessed using the Quality Assessment of Diagnostic Accuracy Studies (QUADAS-2). Funnel plots and Egger’s test were used to evaluate publication bias. The bivariate random-effect model was used for calculating the sensitivity, specificity, positive likelihood ratio, negative likelihood ratio, diagnostic odds ratio, and area under the curve (AUC). A Fagan nomogram was applied to evaluate the clinical applicability of the A2DS2 scale.

**Results:**

A total of 29 full-text articles met the inclusion criteria, including 19,056 patients. Bivariate mixed-effects regression models yielded a mean sensitivity of 0.78 (95 % CI: 0.73–0.83), a specificity of 0.79 (95 % CI: 0.73–0.84), a positive likelihood ratio of 3.7 (95 % CI: 2.9–4.6), and a negative likelihood ratio of 0.27 (95 % CI: 0.23–0.33). The area under the receiver operating characteristic curve was 0.85 (95 % CI: 0.82–0.88). If given a pre-test probability of 50 %, the Fagan nomogram showed that when A2DS2 was positive, the post-test probability improved to 79 %. In contrast, when A2DS2 was negative, it decreased to 22 %. The results of the subgroup analysis showed no effect on the diagnostic accuracy of the A2DS2 scale in predicting stroke-associated pneumonia, except for the optimal cut-off value.

**Conclusions:**

The A2DS2 scale demonstrates high clinical applicability and could be a valid scale for the early prediction of stroke-associated pneumonia in stroke patients.

## Background

Stroke-associated pneumonia (SAP) is a common medical complication of stroke that affects 5.60–37.98 % of stroke patients [1]. Delays in the diagnosis or treatment of SAP increase disease mortality, prolong the length of hospital stay, and increase medical costs. Therefore, it is necessary to find an early and reliable method to predict the risk of SAP. However, previous research usually focuses on diagnosing pneumonia or stroke rather than SAP prediction [2].

In recent years, various risk factors for SAP have been reported. SAP risk factors include mechanical ventilation, atrial fibrillation, pre-existing respiratory disease, smoking, pre-existing heart disease, and dysphasia [3]. Combined with the SAP risk factors, some researchers in different regions established several early prediction SAP scales, such as the A2DS2 scale and the PANTHERS scale in Germany, the ISAN scale in Britain, and the AIS-APS scale in China [4–7]. Indeed, potential deficiencies existed among the different scales, such as their use of various sources, the need for continuous external validation, and their complexity. The A2DS2 scale predicts SAP better than other platforms [8], however, the clinical adaptability of the A2DS2 scale has not been comprehensively and systematically analysed until now.

The A2DS2 scale is one of the most widely used rankings and has been extensively used in the clinic. The A2DS2 scale was developed by Hoffmann et al. and was based on a clinical scale for evaluating SAP after stroke in 15,335 stroke patients in Germany [4]. Multivariate analysis was used to analyse its risk factors and to assign points to form the A2DS2 scale. In this study, the A2DS2 scale had a high sensitivity (83 %) and specificity (72 %). However, another study conducted in China reported that the sensitivity and specificity of the A2DS2 scale were 69 % and 73 %, respectively [9]. Other studies also reported different sensitivities and specificities, which may be related to race, incidence, research methods, and the optimal cut-off value of the scale. Therefore, quantifying and comparing the clinical differences of trials within a meta-analysis is crucial to determining the applicability and use of the ASD2 scale in clinical practice.

This meta-analysis provides evidence for the evaluation and diagnosis of SAP by the A2SD2 scale and verifies its clinical applicability. Appropriate interventions can be combined with predictive outcomes to reduce the risk of SAP.

## Methods

### Data sources and search strategies

Systematic retrieval of literature up to June 2020 in PubMed, Embase, the Cochrane Library, Web of Science, the China National Knowledge Infrastructure database, and the Wanfang database was conducted. The following search terms were used in combination: (( “strokes” OR “cerebrovascular accident” OR “brain vascular accident” OR “apoplexy” ) AND ( “stroke-associated pneumonia” OR “experimental lung inflammation” )) AND ( “A2DS2 scale” OR “score” ).

### Inclusion criteria

The inclusion criteria were as follows: (1) prospective or retrospective clinical studies; (2) patients diagnosed with stroke; (3) patients met the diagnostic criteria for pneumonia [10–12] and stroke [13]; and (4) the true positive value, false positive value, true negative value and false negative value of the A2DS2 scale could be obtained directly or indirectly to predict pneumonia in patients with stroke.

### Exclusion criteria

The exclusion criteria were as follows: (1) studies reporting duplicated results; (2) other scales for predicting SAP; (3) studies with low quality or unavailable data; (4) studies with a gold-standard diagnosis; and (5) reviews, letters, editorials, letters, and case reports.

### Data extraction

Two researchers extracted data independently, including the first author, year of publication, population, patient diagnosis standard, research type, sample size, four types of data (true positive (TP), false positive (FP), true negative (TN), and false negative (FN)) and their degrees of sensitivity and specificity.

### Quality assessment

We assessed the quality of the included studies with the QUADAS-2 checklist [14]. The scale included 14 items divided into four parts, including case selection, trial to be evaluated, gold standard, and process cases. The deviation risk level was determined based on the answers to the questions. Eligibility was agreed upon, and any disagreements were resolved through discussion and mutual consensus.

### Statistical analysis

STATA 12.0 was used for statistical analysis. Interstudy heterogeneity was tested using the I^2^ test, with an I^2^ > 75 % denoting heterogeneity. The outcome parameters were overall sensitivity, specificity, positive likelihood ratio (PLR), negative likelihood ratio (NLR), diagnostic odds ratio (DOR), and their corresponding 95 % confidence intervals (CIs) by a random-effects model. Each included study’s sensitivity and specificity were used to plot the summary ROC (SROC) curves and to calculate the area under the SROC curve (AUC). The PLR and NLR were used to calculate the clinical utility of the A2DS2 scale. The diagnostic odds ratio (DOR) was used as the summary measure of diagnostic accuracy. Sensitivity analysis was performed according to the missing data, risk of bias, and sample size to evaluate the robustness of significant statistical heterogeneity. Subgroup analysis demonstrated whether ethnicity, study type and cut-off value affected the diagnostic accuracy. Deeks’ funnel plot and Egger’s test were used to detect publication bias, with *P* < 0.05 indicating publication bias. The clinical applicability of the A2DS2 scale was evaluated by constructing a Fagan nomogram with combined positive and negative likelihood ratios.

## Results

### Search results and quality evaluation

**Fig. 1:**
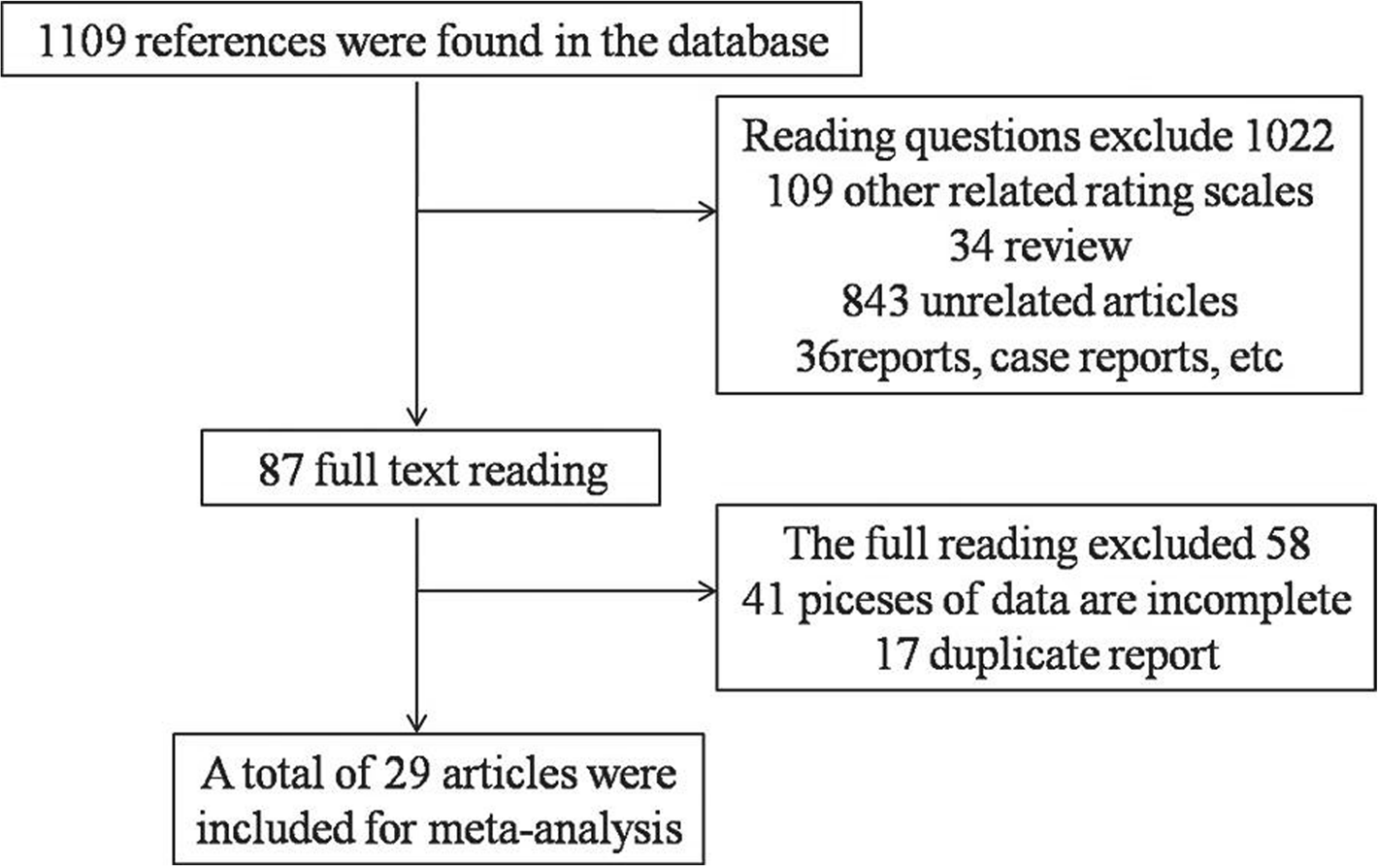
Flow diagram of the study selection process

**Fig. 2:**
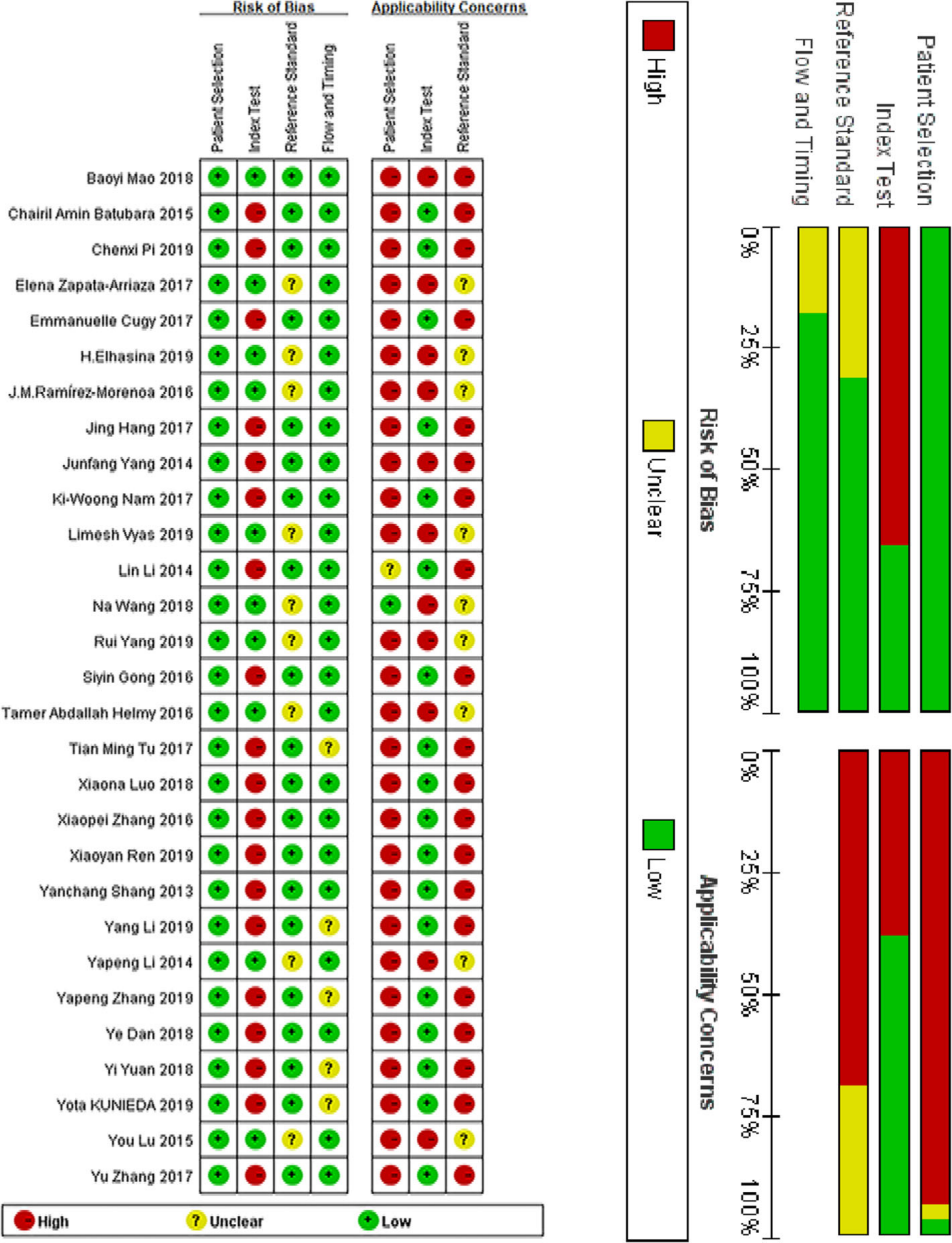
Risk of bias and applicability concerns graph: review of authors’ judgements about each domain, presented as percentages across included studies

Initially, 1569 articles were retrieved; according to the inclusion and exclusion criteria, 29 studies were ultimately included in this study. A screening flowchart of the course is depicted (Fig. 1). Of these 29 studies, 10 were prospective studies, and 19 were retrospective studies. The specific information for each study is shown in Table 1. A total of 19,056 stroke patients were analysed, with the populations coming from different countries. The included subjects had a low risk of migration and high clinical adaptability (Fig. 2). The study characteristics of the data sets are listed in Table 1.

**Table 1:** Baseline characteristics of studies that satisfied the inclusion criteria (TP = true positive, FP = false positive, TN = true negative, FN = false negative)

Author	Region	Year	Type	Sample size	TP	FP	FN	TN	The values of the A2DS2
Yang L [15]	China	2019	retrospective	2552	138	637	62	1715	4
Ren XY [16]	China	2019	retrospective	530	75	89	15	351	5
Yang R [17]	China	2019	prospective	86	16	18	3	49	5
Mao BY [18]	China	2018	prospective	80	44	7	1	28	5
Zhang Y [9]	China	2017	retrospective	2552	162	889	37	1464	3
Pi CX [19]	China	2019	retrospective	215	31	18	13	153	5
Wang N [20]	China	2018	prospective	271	85	15	18	153	5
Hang J [21]	China	2017	retrospective	1472	257	158	138	874	5
Luo XN [22]	China	2018	retrospective	203	27	19	19	138	4.5
Zhang YP [23]	China	2019	retrospective	201	24	27	7	143	7
Shan Y [24]	China	2018	retrospective	252	27	19	20	186	6.5
Yuan Y [25]	China	2018	retrospective	512	61	57	24	427	4
Yang JF [26]	China	2014	retrospective	636	80	64	12	480	7
Zhang XP [1]	China	2016	retrospective	1239	57	174	33	975	5
Shang YC [27]	China	2013	retrospective	131	30	23	8	70	5
Li Y [28]	China	2014	prospective	1142	130	70	85	857	5
Gong SY [29]	China	2016	retrospective	1569	168	455	72	874	3
Lu Y [30]	China	2015	prospective	101	49	17	2	33	5
Li L [31]	China	2014	retrospective	1279	198	78	110	893	10
Batubara CA [32]	Indonesia	2015	retrospective	32	20	6	2	4	5
Limesh V [33]	India	2019	prospective	250	44	71	2	133	5
Tu TM [34]	Singapore	2017	retrospective	731	36	173	4	518	7
Zapata AE [35]	Spain	2017	prospective	201	27	86	4	84	5
Ramírez-Moreno J.M [36]	Spain	2016	prospective	224	30	31	15	209	6
Yota K [37]	Japan	2019	retrospective	111	14	26	3	68	5
Nam KW [2]	Korea	2017	retrospective	299	24	38	18	219	5
Cugy E [38]	French	2017	retrospective	1960	126	365	43	1426	5
Helmy TA [39]	Egypt	2016	prospective	70	20	7	6	37	6
HElhasina [40]	Arab	2019	prospective	200	37	134	5	24	5

### Diagnostic accuracy of the A2DS2 scale

**Fig. 3:**
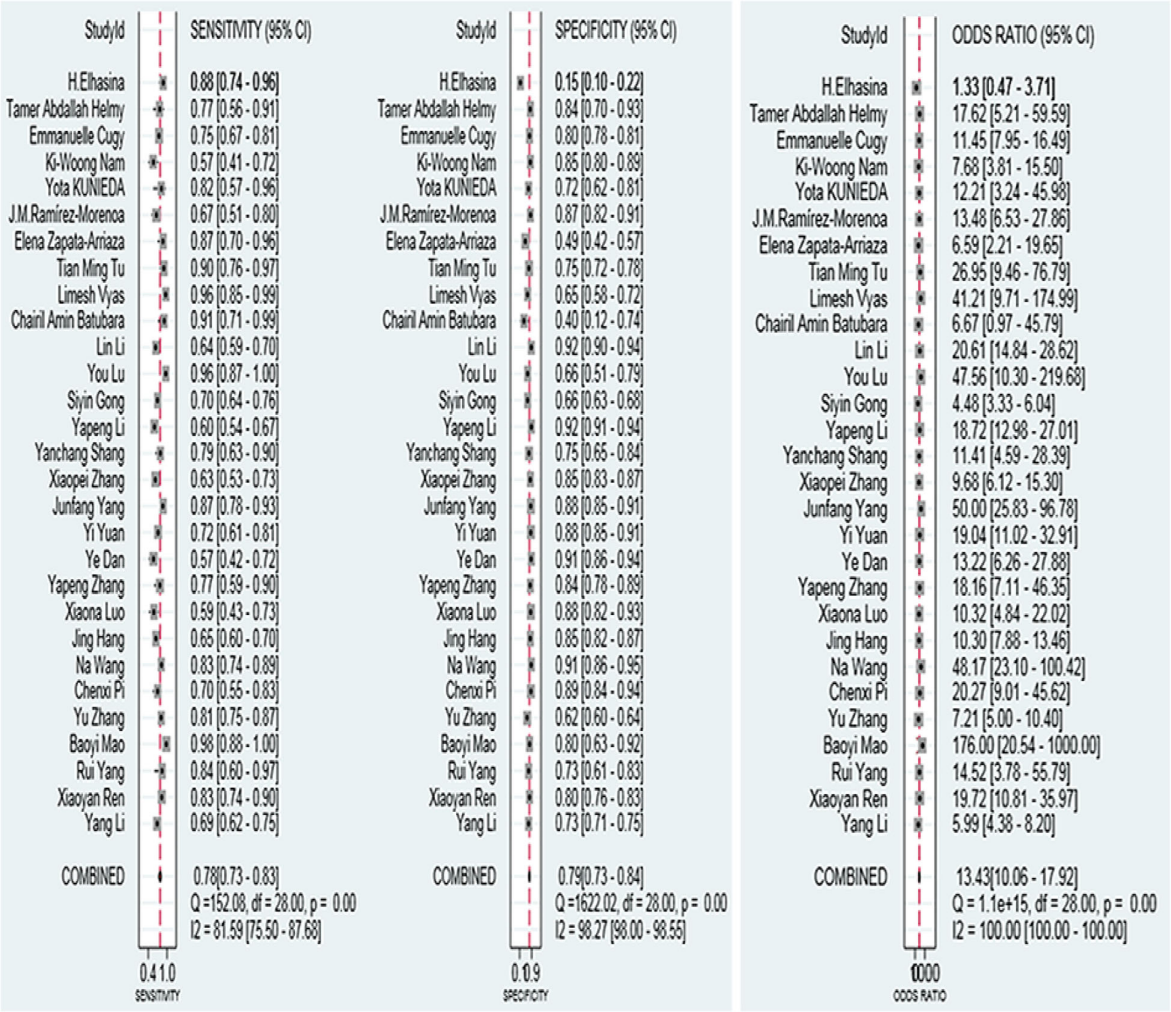
Forest plot: sensitivity, specificity, and DOR of the A2DS2 scale for the diagnosis of SAP

**Fig. 4:**
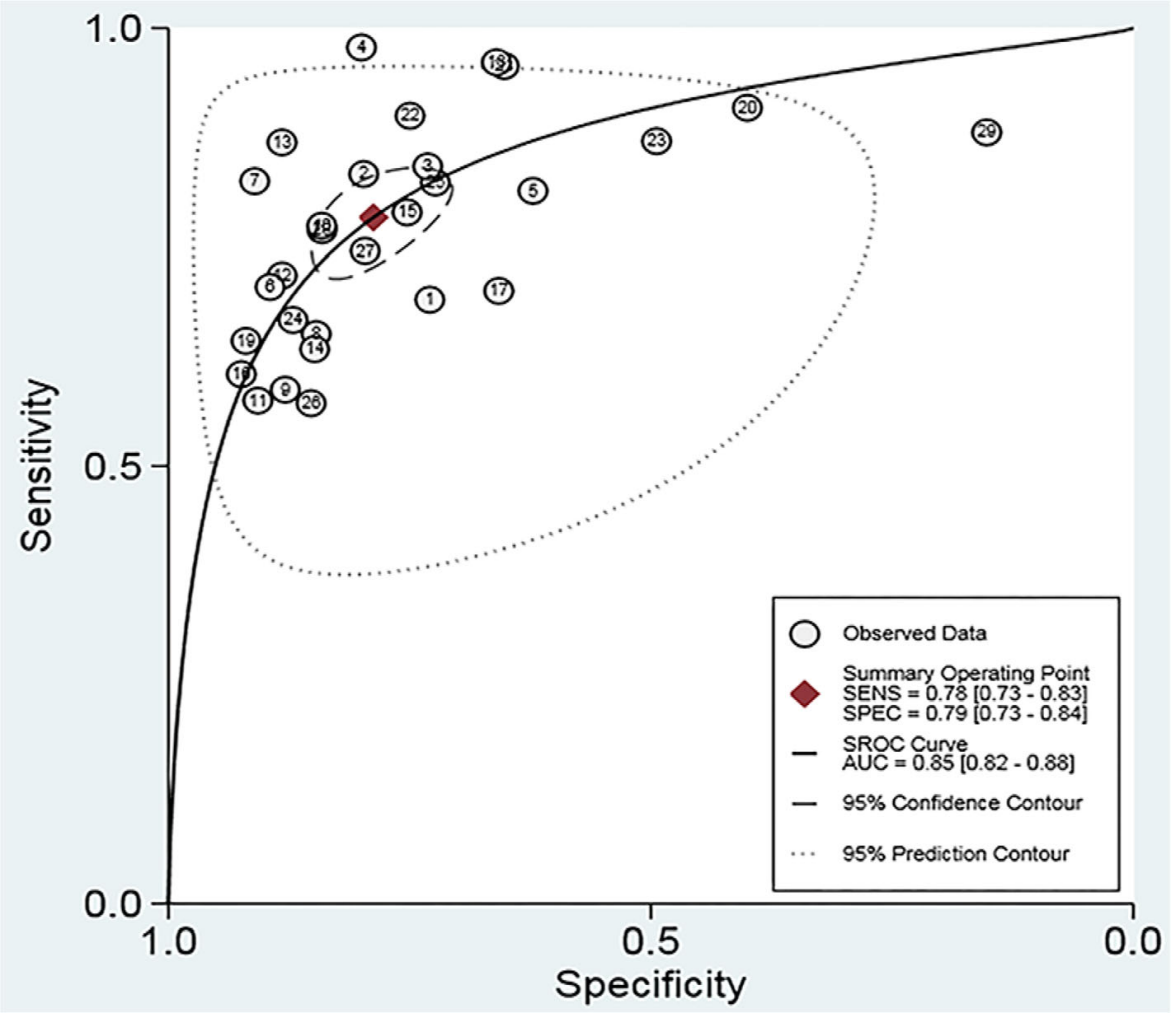
Summary receiver operating characteristic curve showing the 95 % confidence contour and 95 % prediction contour

Meta-analysis showed that the merged sensitivity, specificity, DOR (Fig. 3) and AUC (Fig. 4) of the A2DS2 scale were 0.78 (95 % CI: 0.73–0.83; *P* = 0.00), 0.79 (95 % CI: 0.73–0.84; *P* = 0.00), 13.43 (95 % CI: 10.06–17.92; *P* = 0.00) and 0.85 (95 % CI: 0.82–0.88), respectively.

**Fig. 5:**
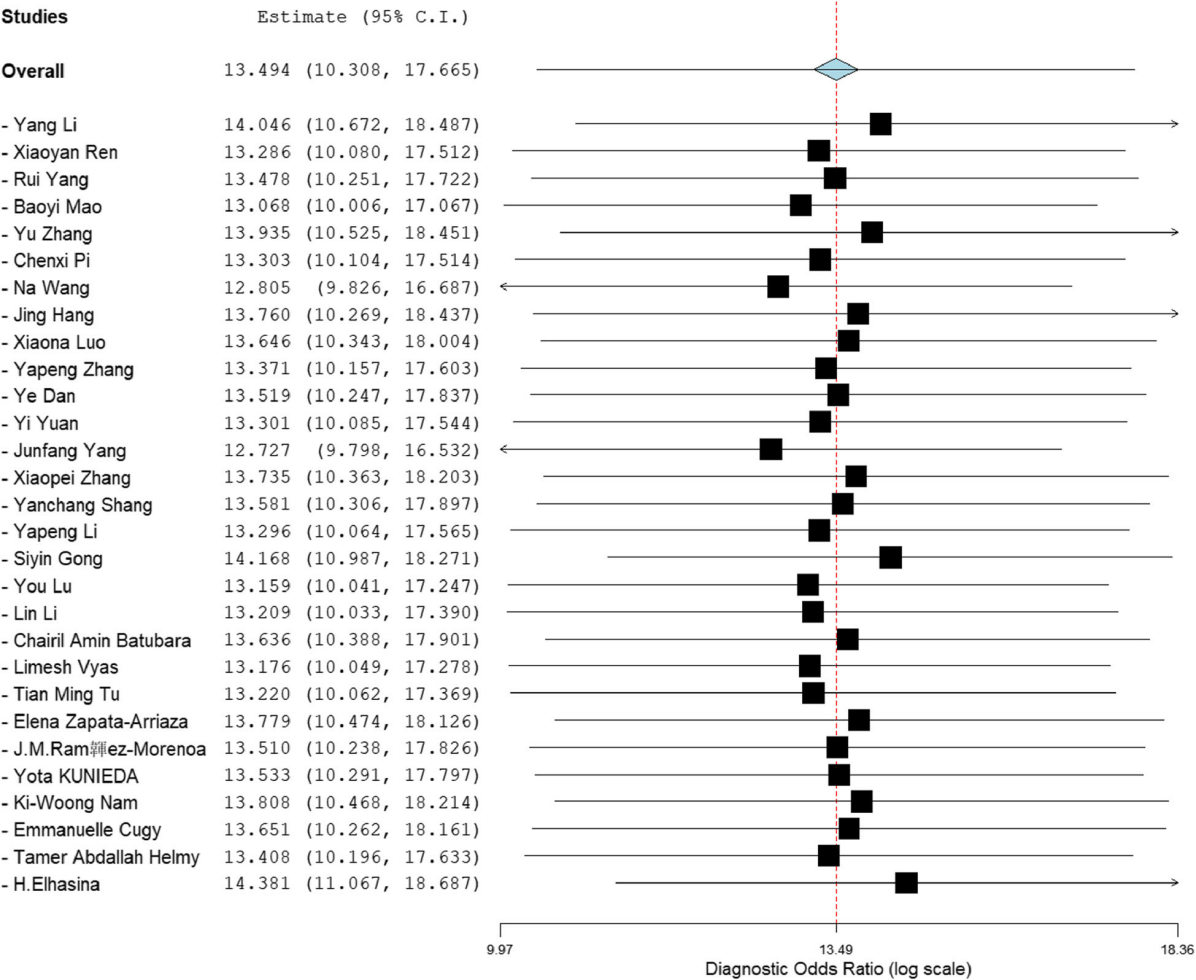
Forest plot: sensitivity analysis among various studies

**Fig. 6:**
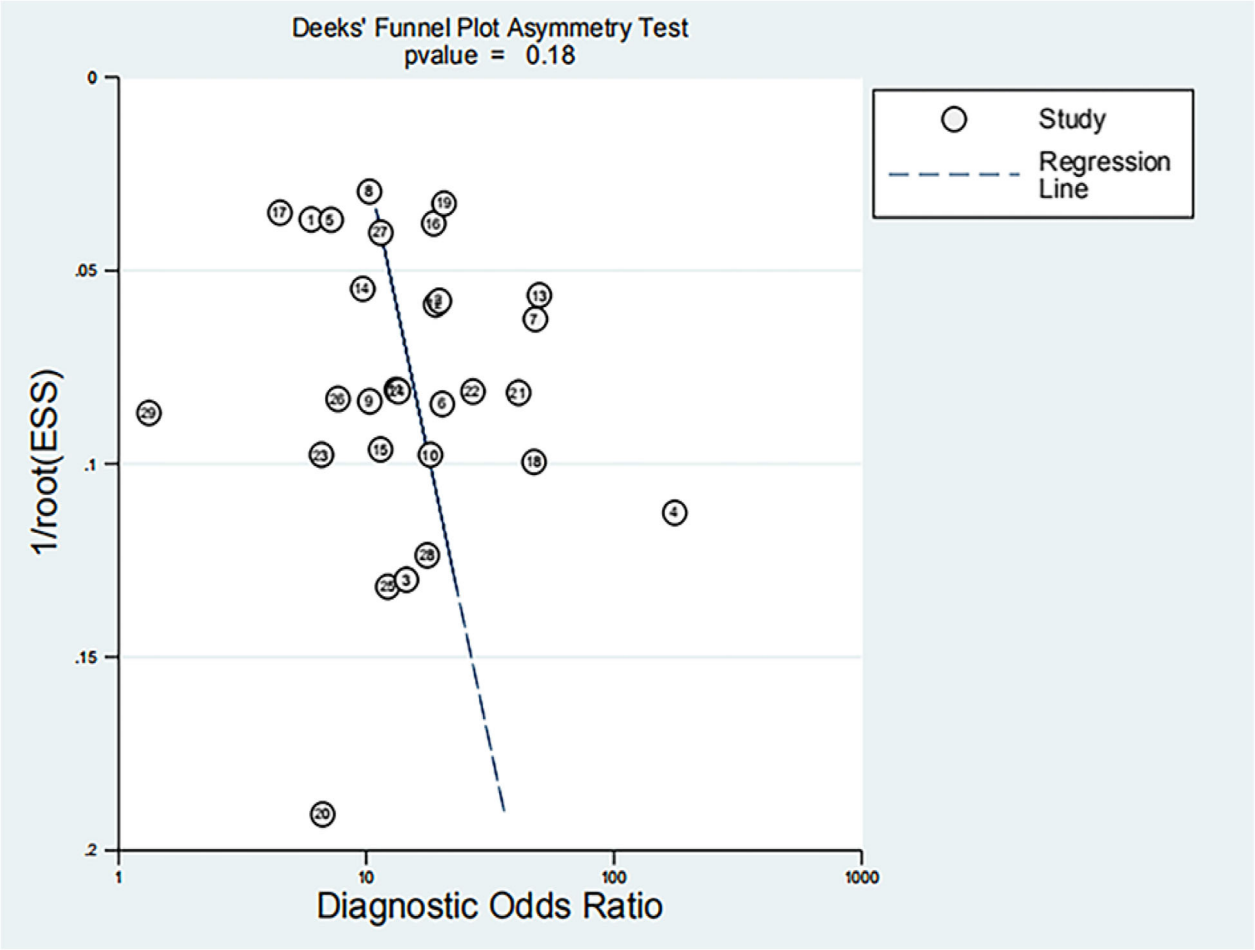
Deeks’ funnel plot: evaluation of article publication bias

**Fig. 7:**
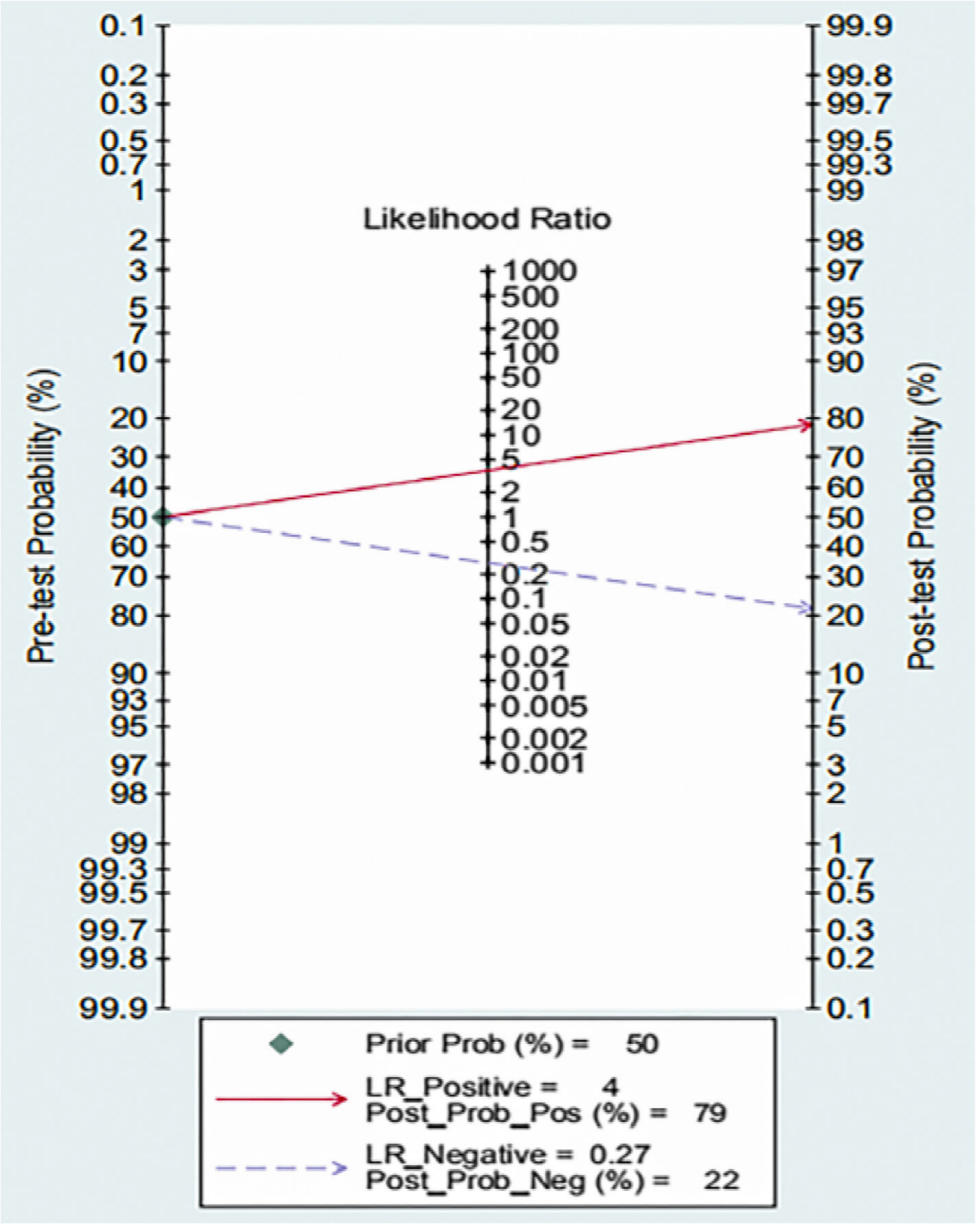
Fagan nomogram of the clinical applicability of the A2DS2 scale for the diagnosis of SAP

According to the sensitivity analysis (Fig. 5), the combined DOR did not change significantly before and after each study was individual excluded, indicating that the study results were stable. The funnel plot showed the symmetry of the studies included in our meta-analysis (Fig. 6). The *p*-value of Egger’s test was 0.19, indicating that there was no publication bias in the study. Fagan nomogram analysis showed that the preset a priori probability of A2DS2 was 50 %, and the predictive diagnostics of A2DS2 were complimentary. The likelihood of stroke patients being diagnosed with stroke-related pneumonia rose to 79 %, and the negative prediction of A2DS2 fell to 22 %, indicating good adaptability and clinical diagnosis of the A2DS2 scale (Fig. 7).

Subgroup analyses were conducted to determine whether ethnicity, study type and cut-off value were sources of heterogeneity affecting the scale’s diagnostic accuracy. The subgroup analysis results did not affect the diagnostic accuracy of the A2DS2 scale in predicting SAP, except for the cut-off value (Table 2). A cut-off value greater than 5 had a sensitivity of 0.75 (0.64, 0.83) and a specificity of 0.87 (0.80, 0.91). Those with less than 5 points and those with 5 points had sensitivities of 0.71 (0.65,0.77) and 0.79 (0.73,0.84) and specificities of 0.76 (0.670.69,0.83) and 0.76 (0.68,0.82), respectively.

**Table 2:** Meta-analysis of the diagnostic accuracy of A2DS2 in different subgroups

Subgroups		Sensitivity (95 % CI)	Specificity (95 % CI)
Study type	retrospective	0.73(0.68, 0.76)	0.82(0.77, 0.85)
	prospective	0.76(0.72, 7.09)	0.79(0.74, 0.83)
Ethnic	Caucasian	0.75(0.65, 0.83)	0.75(0.53, 0.89)
	Asian	0.76(0.71, 0.80)	0.79(0.75, 0.84)
Cut-off value	<5	0.71(0.65, 0.77)	0.76(0.69, 0.83)
	=5	0.79(0.73, 0.84)	0.76(0.68, 0.82)
	>5	0.75(0.64, 0.83)	0.87(0.80, 0.91)

## Discussion

In recent years, some successful management and treatments of acute stroke have emerged. However, stroke remains the leading cause of death worldwide, among which stroke with complications of pneumonia is a critical cause of increasing mortality [41]. It is necessary to assess SAP risk at an early stage; unfortunately, the early diagnosis standards are still not unified. Commonly used scales include the A2DS2 scale, the PANTHERIS score, the ISAN score and the AIP-APS score, but the PANTHERIS score [5] and AIP-APS score [7] require laboratory test results, which further increases the difficulty in the early evaluation of SAP. Researchers have indicated that the A2DS2 scale has a higher c-statistic value than other scales, though its external validation method and specific clinical efficacy need to be verified.

The results of this meta-analysis showed that the AUC of the A2DS2 scale was 0.85 (95 % CI: 0.82, 0.88) for predicting stroke-associated pneumonia, and the sensitivity and specificity of the A2DS2 scale were 0.78 (95 % CI: 0.73, 0.83; *P* = 0.00) and 0.79 (95 % CI: 0.73, 0.84; *P* = 0.00), respectively. The study results suggest that the A2DS2 scale has good diagnostic test accuracy and good potency for SAP prognosis. The A2DS2 scale can be used to assess stroke patients’ status at an early stage and can distinguish those at high risk of developing stroke-associated pneumonia so that early prophylaxis against stroke-associated pneumonia can be given to high-risk patients. Moreover, the scale can help reduce mortality, the length of stay, and hospital costs in stroke patients. Therefore, the A2DS2 scale can be a feasible clinical tool for the early prediction of stroke-associated pneumonia just before laboratory and imaging evaluations.

In 13 of the 29 studies, the sensitivity was higher than the combination sensitivity, and in 17 studies, the specificity was higher than the combination specificity. No exceptions were made for factors such as ethnicity, study type, and cut-off value. The optimal intercept value of the A2DS2 scale in most studies was 5, while this value in several studies was 4 [15] or 6 [16]. Changes in the optimal intercept values, such as taking a larger fraction, will reduce its sensitivity and increase the specificity of the A2DS2 scale, while taking a smaller fraction will reduce its specificity and increase the sensitivity. The most appropriate intercept value, which can increase the diagnostic rate for patients with SAP, remains unclear.

However, in the meta-analysis, the subgroup analysis results confirmed the sensitivity and specificity of the region, study type, sample size, and cut-off value. Subgroup analyses were not significant, except for the cut-off value. The sensitivity and specificity were higher when the cut-off value was equal to 5. Therefore, these results suggest that the A2DS2 scale is a potential prognostic tool that could help clinicians make appropriate treatment decisions and predict the clinical outcome of patients with stroke, especially when the cut-off value is equal to 5. Sensitivity analysis showed that the merged DOR did not change significantly, which means that the external diagnostic accuracy of the A2DS2 scale was verified. Combined with Fagan nomogram analysis, it was verified that the A2DS2 scale was of high clinical value in diagnosing stroke-associated pneumonia.

Our meta-analysis has several limitations. First, the study’s heterogeneity was high (I^2^ > 75 %) due to the incompleteness of data, scale usage time, and optimal cut-off value. Second, despite its high sensitivity and specificity, the A2DS2 scale does not entirely exclude false-positive and false-negative rates. Therefore, the A2DS2 scale can be considered only as an SAP screening tool and not a diagnostic tool. The inclusion of those studies may weaken the diagnostic accuracy of the survey to some extent. Third, some high-quality studies were excluded because complete data could not be obtained, and their extensive external adaptability needs to be further verified.

## Conclusions

The A2DS2 scale is a feasible and straightforward scale for the early screening of SAP, with high external adaptability. A prospective multiregional and large-sample study of stroke patients is still needed to verify our results.

## Data Availability

All data generated or analysed during this study are included in this published article.

## Abbreviations

QUADAS: Quality Assessment of Diagnostic Accuracy Studies
AUC: Area under the curve
CI: Confidence interval
SAP: Stroke-associated pneumonia
TP: True positive
FP: False positive
TN: True negative
FN: False negative
PLR: Positive likelihood ratio
NLR: Negative likelihood ratio
DOR: Diagnostic odds ratio
SROC: Summary receiver operating characteristic curve
